# Identification and Validation of Loci Governing Seed Coat Color by Combining Association Mapping and Bulk Segregation Analysis in Soybean

**DOI:** 10.1371/journal.pone.0159064

**Published:** 2016-07-12

**Authors:** Jian Song, Zhangxiong Liu, Huilong Hong, Yansong Ma, Long Tian, Xinxiu Li, Ying-Hui Li, Rongxia Guan, Yong Guo, Li-Juan Qiu

**Affiliations:** The National Key Facility for Crop Gene Resources and Genetic Improvement (NFCRI) and MOA Key Lab of Soybean Biology (Beijing), Institute of Crop Science, Chinese Academy of Agricultural Sciences, Beijing, P. R. China; National Institute of Plant Genome Research (NIPGR), INDIA

## Abstract

Soybean seed coat exists in a range of colors from yellow, green, brown, black, to bicolor. Classical genetic analysis suggested that soybean seed color was a moderately complex trait controlled by multi-loci. However, only a couple of loci could be detected using a single biparental segregating population. In this study, a combination of association mapping and bulk segregation analysis was employed to identify genes/loci governing this trait in soybean. A total of 14 loci, including nine novel and five previously reported ones, were identified using 176,065 coding SNPs selected from entire SNP dataset among 56 soybean accessions. Four of these loci were confirmed and further mapped using a biparental population developed from the cross between ZP95-5383 (yellow seed color) and NY279 (brown seed color), in which different seed coat colors were further dissected into simple trait pairs (green/yellow, green/black, green/brown, yellow/black, yellow/brown, and black/brown) by continuously developing residual heterozygous lines. By genotyping entire F_2_ population using flanking markers located in fine-mapping regions, the genetic basis of seed coat color was fully dissected and these four loci could explain all variations of seed colors in this population. These findings will be useful for map-based cloning of genes as well as marker-assisted breeding in soybean. This work also provides an alternative strategy for systematically isolating genes controlling relative complex trait by association analysis followed by biparental mapping.

## Introduction

Soybean [*Glycine max* (L.) Merr.] is the most widely grown grain legumes in the world, which is widely used as the major sources of vegetable oils and plant proteins [1]. Soybean seed contains eight essential amino acids which could not be produced by human body [2]. Seed coat color is an important attribute determining outward appearance of soybean seed, which exists in a range of colors from yellow, green, brown, black, to bicolor. It is usually considered as a useful phenotypic marker in breeding due to convenience for observation [3, 4]. Compared with yellow seeds of most grown soybean varieties, black/brown seeds usually accumulate flavonoids and anthocyanins within the epidermal layer of the seed coat, which are currently attracting great interest in their antioxidant properties and flavors [5]. Seed coat color is also an evolutionary trait within the *soja* subgenus and it was changed from black in wild soybean to various colors in cultivated soybeans during domestication [6, 7]. In addition, several studies have also concerned partial pigmentation of seed coat as a result of chilling stress or viral diseases, indicating crosstalk between regulation of seed coat pigmentation and stress responses [8–13].

Soybean seed color has moderately complex inheritance which is controlled by multi-loci. At least five genetic loci (*I*, *R*, *T*, *W1*, and *O*) were identified by classical genetics, most of which were involved in flavonoid-based pigmentation pathway [13, 14]. Among them, three (*I*, *R*, and *T*) are involved in the biosynthesis of the pigments while *O* and *W1* only influence the pigmentation under the background of recessive alleles of *i r* or *i t*, respectively [14]. There are four alleles (known as *I*, *i*^*i*^, *i*^*k*^, and *i*) at *I* locus controlling the presence/absence and spatial distribution of anthocyanin and proanthocyanidin *via* posttranscriptional gene silencing. Soybeans possessing dominant *I* allele exhibit complete colorless of seed coat while soybeans with *i* allele give rise to colored seed coat [12]. The other two alleles (*i*^*i*^ and *i*^*k*^) restrict pigments to the hilum and saddle regions of the seed coat [14]. *R* and *T* loci control the type and abundance of pigments in seed coat, resulting in specific colors including black (*i*,*R*,*T*), imperfect black (*i*,*R*,*t*), brown (*i*,*r*,*T*), or buff (*i*,*r*,*t*) [15, 16]. *W1* locus only affects seed color under *iRt* background and *W1* and *w1* alleles give imperfect black and buff colors, respectively. *O* locus affects color of brown seed and soybeans with the recessive *o* allele under *irT* background exhibit red-brown seed coat [14]. In addition, mutants with different combinations (single, double or triple mutants) of *G*, *d1* and *d2* loci give rise to green seed color and segregation of *G1*, *G2*, and *G3* for green color has also been studied previously [17–19].

Molecular cloning of these loci suggested that many of them were structural or regulatory genes involving in anthocyanin biosynthesis pathway. *I* locus was mapped to a region harboring a cluster of chalcone synthase (CHS) genes on chromosome 8 of soybean genome [20–22]. The recessive *i* allele had a deletion of *CHS4* or *CHS1* promoter sequences, resulting in an increased accumulation of chalcone synthase (CHS) transcripts in the seed coat due to the abolishment of posttranscriptional RNA silencing [23, 24]. Cloning of genomic and cDNA sequences of flavonoid 3’-hydroxylase (*F3’H*) gene suggested that this gene cosegregated with *T* locus [25, 26]. Chromatographic experiments and genetic analysis also revealed that *W1* might encode a flavonoid 3’ 5’ hydroxylase (F3’5’H) as a 65-bp insertion in this gene cosegregated with the mutant phenotype [15, 27]. *R* locus was initially mapped to LG K (chromosome 9) [28] and then restricted to a region between molecular markers A668_1 and K387_1 [29]. Candidate gene analysis suggested that loss function of a seed coat-expressed *R2R3-MYB* gene was responsible for recessive phenotype of *R* locus [30, 31]. Furthermore, *O* locus has been found to correspond to an anthocyanidin reductase (ANR) gene, which needs to be further confirmed [13]. Recently, cloning and characterization of *D1* and *D2* revealed that they were homologs of the *STAY-GREEN* (*SGR*) genes from other plant species and were duplicated as a result of the most recent whole genome duplication in soybean [32, 33].

Both biparental and association mapping are two main approaches for genetic dissection of important traits in plants [34]. Traditionally, biparental mapping served as a powerful tool to identify genes for QTLs in model plants *Arabidopsis* and rice [35–41]. In the subsequent processes of positional cloning, the most effective way for characterization of individual locus is the use of near isogenic lines (NILs) which differ only at a single QTL region. However, it still has limitations in isolation of genes for QTLs in plants with complex genome such as soybean, which is mainly due to limited allelic diversity existing in two parental lines and low recombination events incurring during population development. Especially, development of NILs through repeated backcrossing is still a time-consuming and laborious process for soybean. Therefore, only a few reports have been published in successful isolation of genes responsible for QTLs in soybean [42, 43]. Alternatively, association mapping using natural population has also proven to be an effective strategy to identify marker-trait associations in animals and plants [44]. Association mapping enables the study of many genotypes at once and generates more precise QTL positions if a sufficient number of molecular markers are used. Therefore, this mapping method has been shown to have potential in dissecting the genetic basis of various traits in *Arabidopsis*, rice, and maize [45–47]. However, no correction for multiple testing possibly led to false positive associations [48]. The development of high-throughput sequencing technologies provides the opportunity to combine these two approaches together, which mitigates each other's limitations [49–51].

Classical genetic analysis demonstrated that multi-loci controlled seed coat color in soybean, accessions possessing the same color possibly having different genotypes at these loci. In this study, association mapping coupled with biparental mapping were employed to systematically dissect genes/loci controlling seed coat color of soybean. SNPs in coding regions among 56 soybean accessions were selected for association mapping and a total of 14 genomic regions were identified to be associated with seed coat color. A segregating population derived from two accessions with different colors was used to confirm association mapping results. The inheritance of seed color in this biparental population was dissected into simple color pairs by development of residual heterozygous lines (RHLs). All four loci governing this trait were systematically identified by bulk segregation analysis (BSA) and fine mapping. All these results suggested that association mapping combined with BSA in biparental population acted as a useful strategy for dissecting relative complex traits in soybean, thus providing a valuable tool for marker-assisted breeding.

## Materials and Methods

### Plant materials

For association mapping, a panel of 56 accessions including *G*. *soja* and *G*. *max* were used, which were resequenced in the previous reports [7, 52]. Among them, 21 wild soybeans and three landraces have black seed coat color while four wild soybeans and four landraces have brown. Seed coats of the other five landraces and all 20 breeding lines are yellow (S1 Table). The segregating population consisting of 171 lines was derived from the cross between ZP95-5383 (yellow seed coat) and NY279 (brown seed coat). RHLs were developed by phenotypic selection and self-fertility of specific lines for several generations.

### Genotypic data analysis

SNP data of all 56 accessions were downloaded from NCBI web site (http://www.ncbi.nlm.nih.gov/ SNP/snp_viewTable.cgi?handle = NFCRI_MOA_CAAS). Three sets of SNPs (Set A, B, and C) were selected from entire data set. These sets include SNPs appeared in coding regions (Set A), coding SNPs removal of synonymous ones (Set B) and non-synonymous coding SNPs (Set C). The number of alleles and the polymorphism information content (PIC) per locus were calculated using POWERMAKER 3.25 software [53]. The population structure was assessed by using STRUCTURE software version 2.2 [54]. To determine the number of genetic clusters (*K*), ten independent runs were carried out for each value of *K* (from 1 to 10) with 500,000 iterations, followed by a burn-in period of 500,000 iterations. The likely number of sub-populations present was estimated following Evanno et al.[55], in which the number of sub-groups (∆k) was maximized. The Q matrix that lists the estimated membership coefficients of individuals in each cluster was utilized for subsequent association mapping.

### Association mapping

TASSEL 3.0 software package was used to conduct association mapping and identify associated SNPs with MLM model (Q+K) [48, 56]. Population structure (Q) and the kinship matrix (K) were based on the results of population structure analysis. All SNP-trait pairs with *P*-value < 0.001 were considered significant, which was determined according to the result of QQ-Plot analysis. QQ plots and manhattan plots for association mapping were drawn using the qqman R package [57]. The genotypes of most significant associated SNPs in different soybean accessions were examined using GGT software [58].

### DNA isolation

Genomic DNA was isolated from fresh young leaves of soybeans using the sodium dodecyl sulphate (SDS) method [59, 60]. The extracted DNA was quantified using Quawell Q5000 spectrophotometer (Quawell Technology, Inc. USA) and all DNA samples were normalized to 50ng/μL for PCR amplification.

### Molecular marker analysis

Polymorphic SSR markers in specific mapping regions were developed using parental lines of the segregating population and the progeny were genotyped as previously described [61]. Primer sequences of SSR markers were obtained from SoyBase (http://soybase.org/resources/ssr.php) and Song et al. [62]. PCR was performed in a 20μL reaction system using 1μL of DNA sample in each reaction and conducted in a PTC-200 thermocycler (Bio-Rad, USA).

### Bulk segregation analysis and fine mapping

Residual heterozygous lines with separation of different seed color pairs were used for rough mapping. DNA samples isolated from 20 plants with dominant trait and 20 plants with recessive traits from each RHL population were pooled together to construct two bulks for BSA, respectively. DNA of parental lines and all bulks was screened with SSR markers near loci identified by association mapping. The physical positions of all markers were according to soybean reference genome assembly v1.1 [63]. Once an associated locus was confirmed in a RHL population, the progeny of this RHL were genotyped with additional polymorphic markers from this genomic region. Based on the exchanges between genotypes of markers and specific locus, the recombinants were identified and used for fine mapping.

### Genetic analysis of different loci

SSR markers closely linked to *qSC1;5;7* loci and dCAPs marker of *qSC2/T* locus [64] were used for genotyping entire F_2_ population. dCAPS marker was developed by artificial introduction of a restriction enzyme recognition site at the end of the forward primer for *GmF3’H* gene [64]. PCR products were digested with restriction enzyme *Eco*NI at 37°C for more than 1h, and separated on 2% agarose gels stained with EB followed by photography. The relationship of genotype and phenotype were applied for genetic analysis of different loci.

## Results

### SNP marker selection and distribution analysis

After filtering from more than 5.1 million high quality SNPs identified by combining resequencing data of 31 and 25 soybean accessions [7, 52], three sets of SNPs located in coding regions were selected. There are 176, 065 SNPs in Set A, which appear in coding regions of predicted genes and represent coding SNPs. Set B (98,244 SNPs) represents SNPs removal of synonymous coding SNPs from Set A, including non-synonymous, nonsense and read through coding SNPs. Set C contains 94,261 SNPs and represents only non-synonymous coding SNPs among all 56 accessions (Table 1).

**Table 1:** Distribution of coding SNPs selected from resequencing data.

Chr.	Length (Mb)	No. of SNPs	SNPs/kb	No. of predicted genes	Set A (Coding SNPs)^a^		Set B (Non-synonymous, nonsense and read through coding SNPs)^b^		Set C (Non-synonymous coding SNPs)^c^	
					No. of SNPs	SNPs/Gene	No. of SNPs	SNPs/Gene	No. of SNPs	SNPs/Gene
1	55.92	268,724	4.8	2,428	7681	3.2	4374	1.8	4186	1.7
2	51.66	234,553	4.5	3,158	8495	2.7	4644	1.5	4463	1.4
3	47.78	282,502	5.9	2,641	9145	3.5	5115	1.9	4913	1.9
4	49.24	240,026	4.9	2,575	7258	2.8	3984	1.5	3842	1.5
5	41.94	184,474	4.4	2,636	7139	2.7	3872	1.5	3741	1.4
6	50.72	294,307	5.8	3,296	10797	3.3	6112	1.9	5898	1.8
7	44.68	235,011	5.3	2,801	8916	3.2	4980	1.8	4807	1.7
8	47.00	266,154	5.7	3,867	11465	3.0	6278	1.6	6052	1.6
9	46.84	257,758	5.5	2,729	8583	3.1	4732	1.7	4574	1.7
10	50.97	247,193	4.8	2,986	8449	2.8	4595	1.5	4428	1.5
11	39.17	179,976	4.6	2,866	7020	2.4	3852	1.3	3717	1.3
12	40.11	192,646	4.8	2,484	7061	2.8	3885	1.6	3753	1.5
13	44.41	277,854	6.3	3,786	10917	2.9	6002	1.6	5817	1.5
14	49.71	238,506	4.8	2,201	7553	3.4	4315	2.0	4161	1.9
15	50.94	320,440	6.3	2,715	9510	3.5	5606	2.1	5390	2.0
16	37.40	251,100	6.7	2,138	10235	4.8	5927	2.8	5694	2.7
17	41.91	223,989	5.3	2,685	7497	2.8	3980	1.5	3831	1.4
18	62.31	412,564	6.6	2,580	12887	5.0	7336	2.8	7045	2.7
19	50.59	254,340	5.0	2,641	7709	2.9	4240	1.6	4078	1.5
20	46.77	240,127	5.1	2,369	7748	3.3	4415	1.9	4231	1.8
Scaffold	23.51	-	-	205	-	-	-	-	-	-
Total	973.58	5,102,244	5.2	55,787	176065	3.2	98244	1.8	94621	1.7

^a^Set A represented coding SNPs in which all SNPs appeared in coding regions of predicted genes.

^b^Set B represented SNPs removal of synonymous coding SNPs from Set A, including non-synonymous, nonsense and read through coding SNPs.

^c^Set C represented only non-synonymous coding SNPs.

The distribution of selected SNPs was fairly uniform across all soybean chromosomes (Table 1). The largest number of coding SNPs was observed on chromosome 18, followed by chromosome 8, and the lowest number of SNPs was found on chromosomes 11 and 12. On average, about 3.2 coding SNPs/gene were selected from 5.2 SNPs/kb for the entire genome. For each chromosome, the distribution of coding SNPs varied from 2.4 SNPs/gene on Chromosome 11 to 5.0 SNPs/gene on chromosome 18 (Table 1).

### Population structure analysis

**Fig 1:**
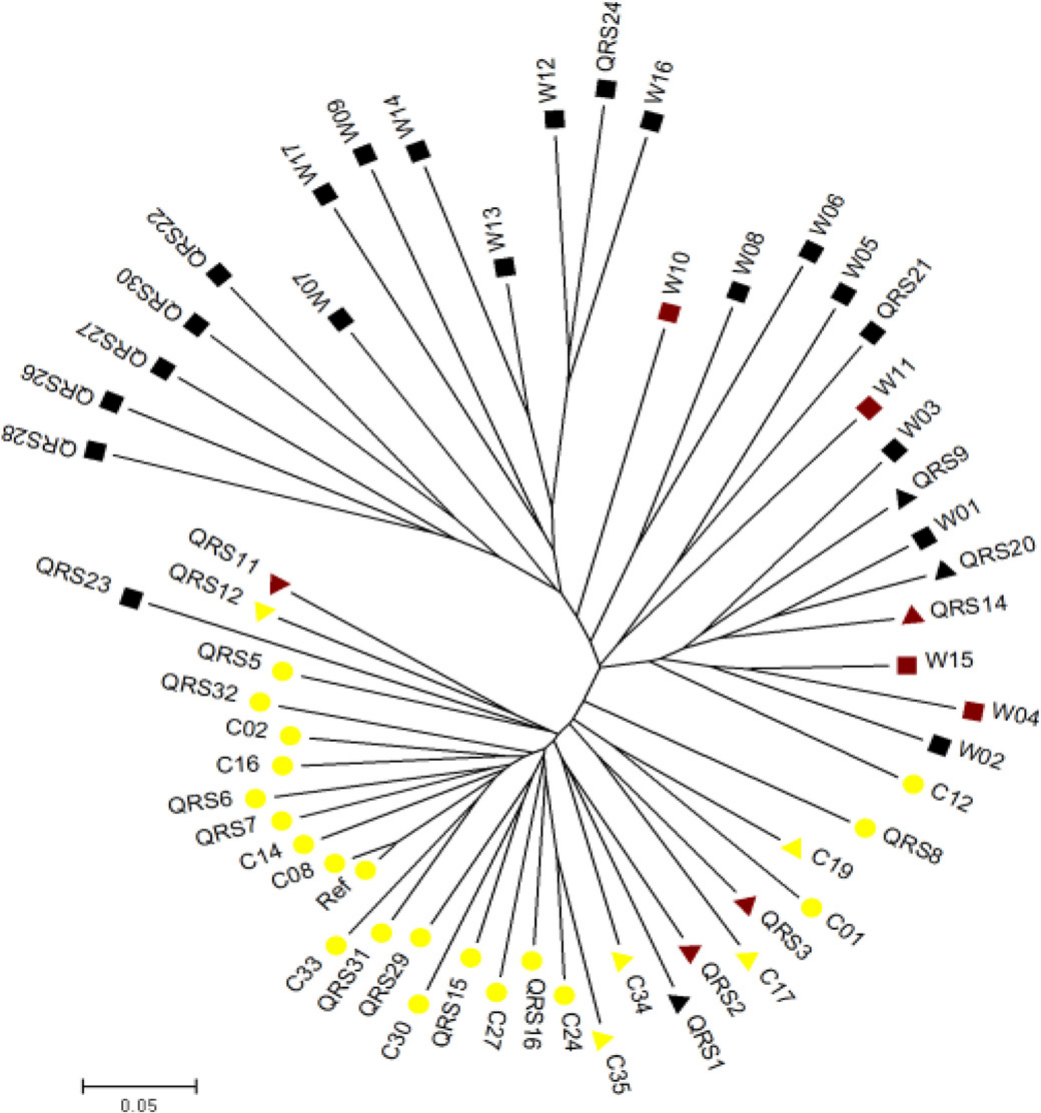
Phylogenetic tree of 56 soybean accessions. The phylogenetic tree was constructed by Powermarker using the coding SNPs. Different shapes indicated different types of accessions (square, wild soybean; triangle, landrace; circle, breeding line) and color of the shape (yellow, brown, and black) indicated seed coat color.

To study the relationship of these 56 soybean accessions, a neighbor-joining tree based on genetic distances was constructed by Powermarker using coding SNPs. The results showed that all these accessions could be classified into two major groups (Fig 1). Majority accessions of *G*. *max* or *G*. *soja* separated completely with only three exceptions (QRS23 in subgroup I mainly containing *G*. *max* and QRS14 and QRS20 in subgroup II mainly containing *G*. *soja*). Meanwhile, population structure was also assessed to estimate the most likely number (*K*) of subgroups among these accessions. The value of LnP(D) increased continuously for *K* values ranging from 1 to 10 and only one significant change of Δ*K* was observed at *K* = 2 (S1 Fig A), suggesting that this natural population could be clustered into two major subgroups (S1 Fig B). Subgroup I included mainly *G*. *max* while subgroup II contained mainly *G*. *soja*, which was in accordance with the neighbor-joining tree (Fig 1).

### Identification of loci associated with seed coat color by association mapping

**Fig 2:**
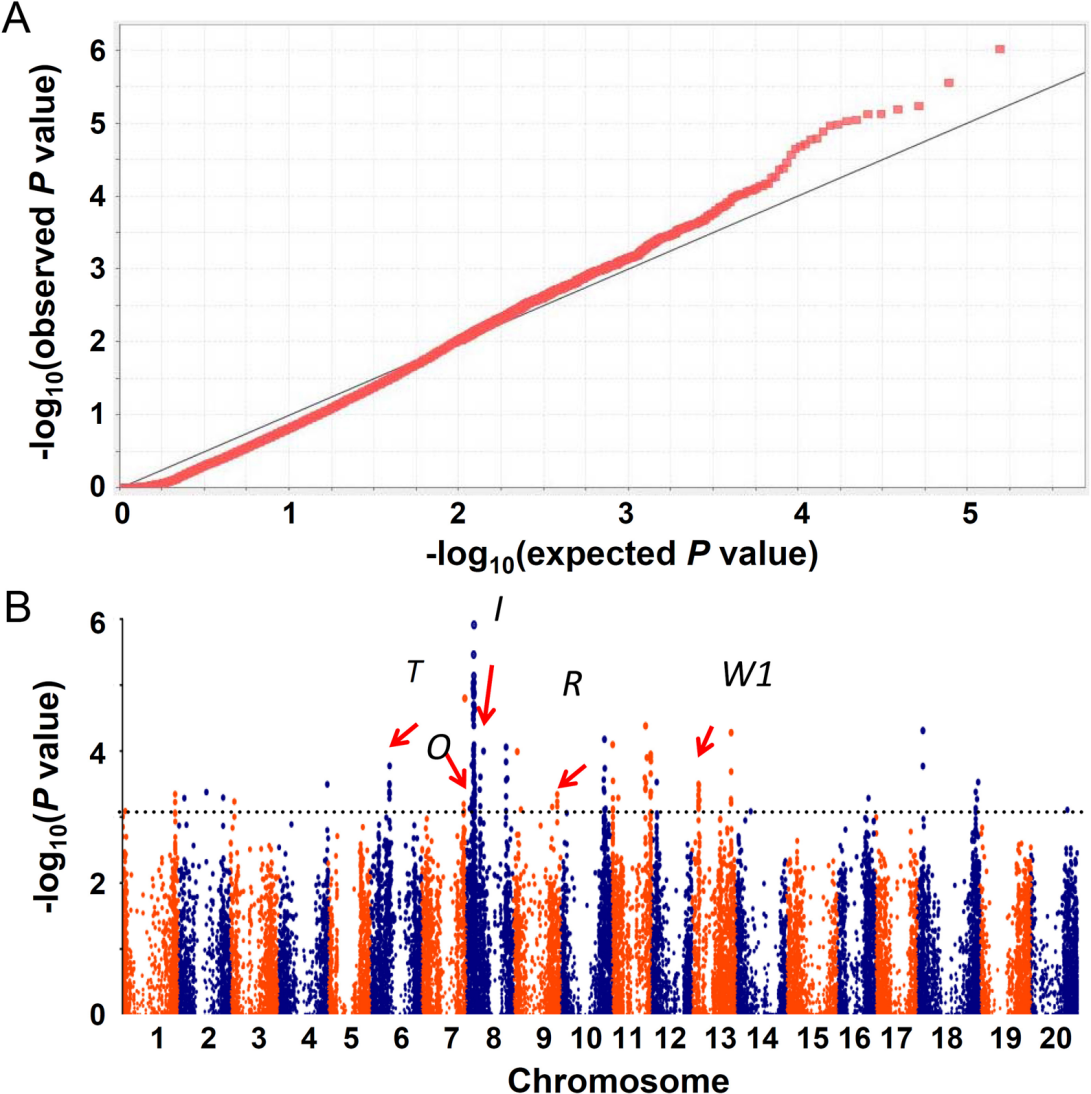
Association mapping of seed coat color in soybean. (A) Expect -log (*P*) matched observed -log (*P*) best from the QQ-Plot. (B) Manhattan plots showed -log (*P*) from a genome-wide scan were plotted against positions of SNPs across 20 chromosomes of soybean. The horizontal line represented threshold of significant association and red arrows indicated the positions of five classical genetic loci.

**Fig 3:**
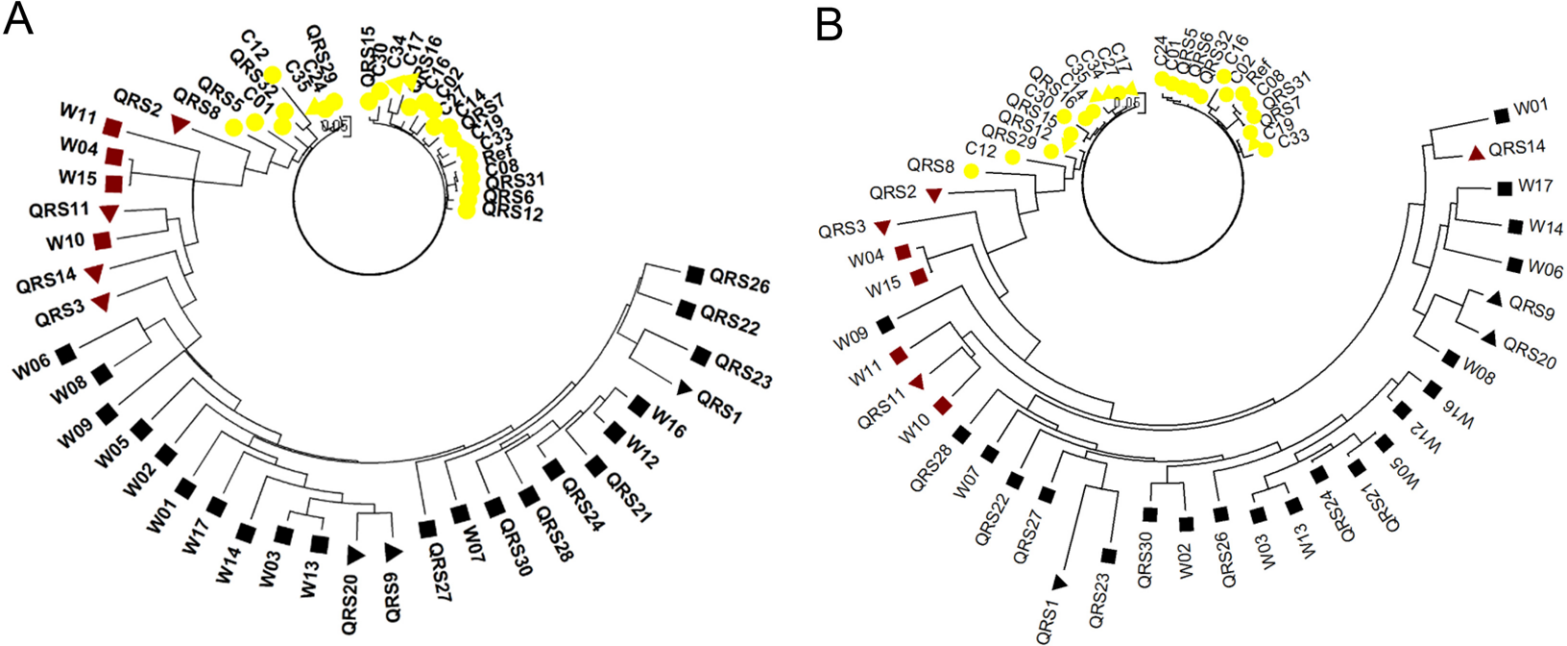
**Phylogenetic tree constructed by Powermarker using associated SNPs (A) and SNPs in five previous reported loci (B).** (A) SNPs located in all 14 associated loci were used for constructing phylogenetic tree and accessions with different seed colors could be separated properly. (B) SNPs in five previous reported loci were used for constructing phylogenetic tree and soybeans with different seed colors can not be separated completely. Different shapes indicated different types of accessions (square, wild soybean; triangle, landrace; circle, breeding line) and color of the shape (yellow, brown, and black) indicated seed coat color.

Association mapping was performed with MLM using the phenotypic data and three sets of SNPs. To reduce both false positive and false negative risks caused by population structure, only SNPs detected by *K* = 2 were taken into account. The QQ-Plot analysis showed that expected -log (*P*) matched observed -log (*P*) best using SNPs from Set A (Fig 2A). Association mapping revealed that 146 SNPs located in 14 genomic regions on 10 chromosomes (designated as *qSC1*-*qSC14*, Fig 2B, Table 2) were significantly associated with seed coat color. Nearly all of 14 regions contained more than five significant associated SNPs except *qSC11* on chromosome 12. The physical distances of these associated regions ranged from 53 kb to 5,142 kb (Table 2). Moreover, similar results were also obtained by using the other two sets (Set B and C) of SNPs (S2 Fig and S2 Table). Interestingly, all five loci identified by classic genetics were detected in our result of association mapping (Fig 2B and Table 2), suggesting the representative of soybean accessions used in this study and the accuracy of mapping result. Furthermore, associated SNPs located in all 14 loci could separate soybeans with different seed colors properly no matter they were wild soybeans, landraces or breeding lines while only SNPs in five previous reported loci could not separate them completely (Fig 3). Even more, the combination of most significant associated SNP in each locus could also identify different seed coat colors of all these accessions (S3 Fig).

**Table 2:** Details of loci associated with seed coat color identified via association mapping.

Locus	Chr.	Position of most significant SNPs	*P*-value	*R*^*2*^	No. of coding SNPs	Significant region			Classic loci
						Start	End	Range(kb)	
*qSC1*	1	52,438,308	3.96E-04	0.2685304	5	51,388,129	52,467,583	1079	-
*qSC2*	6	19,047,336	1.45E-04	0.3022027	7	18,878,314	19,047,336	169	*T*
*qSC3*	7	43,082,019	1.32E-05	0.4644675	6	41,940,392	43,082,019	1142	-
*qSC4*	8	5,501,159	1.42E-04	0.318538	6	3,417,779	5,512,389	2095	*O*
*qSC5*	8	7,589,623	9.56E-07	0.6492089	51	6,783,439	8,648,879	1865	*I*
*qSC6*	8	39,553,339	7.40E-05	0.3314216	8	39,473,028	40,585,746	1113	-
*qSC7*	9	43,418,250	3.55E-04	0.2882047	5	38,277,760	43,419,283	5142	*R*
*qSC8*	10	43,409,021	5.61E-05	0.3567043	15	42,771,760	43,820,987	1049	-
*qSC9*	11	681,423	6.77E-05	0.3912334	9	681,423	1,055,084	374	-
*qSC10*	11	38,426,187	9.55E-05	0.3558796	12	38,370,581	38,636,896	266	-
*qSC11*	12	5,404,396	2.57E-04	0.2872759	3	5,404,396	5,649,464	245	-
*qSC12*	13	7,117,537	2.95E-04	0.271697	9	6,661,165	7,743,106	1082	*W1*
*qSC13*	13	39,050,259	4.40E-05	0.4384943	5	39,045,030	39,157,835	113	-
*qSC14*	18	57,962,686	3.67E-04	0.2844445	5	57,910,138	57,962,686	53	-

### Validation of loci governing seed coat color using bi-parental population

**Fig 4:**
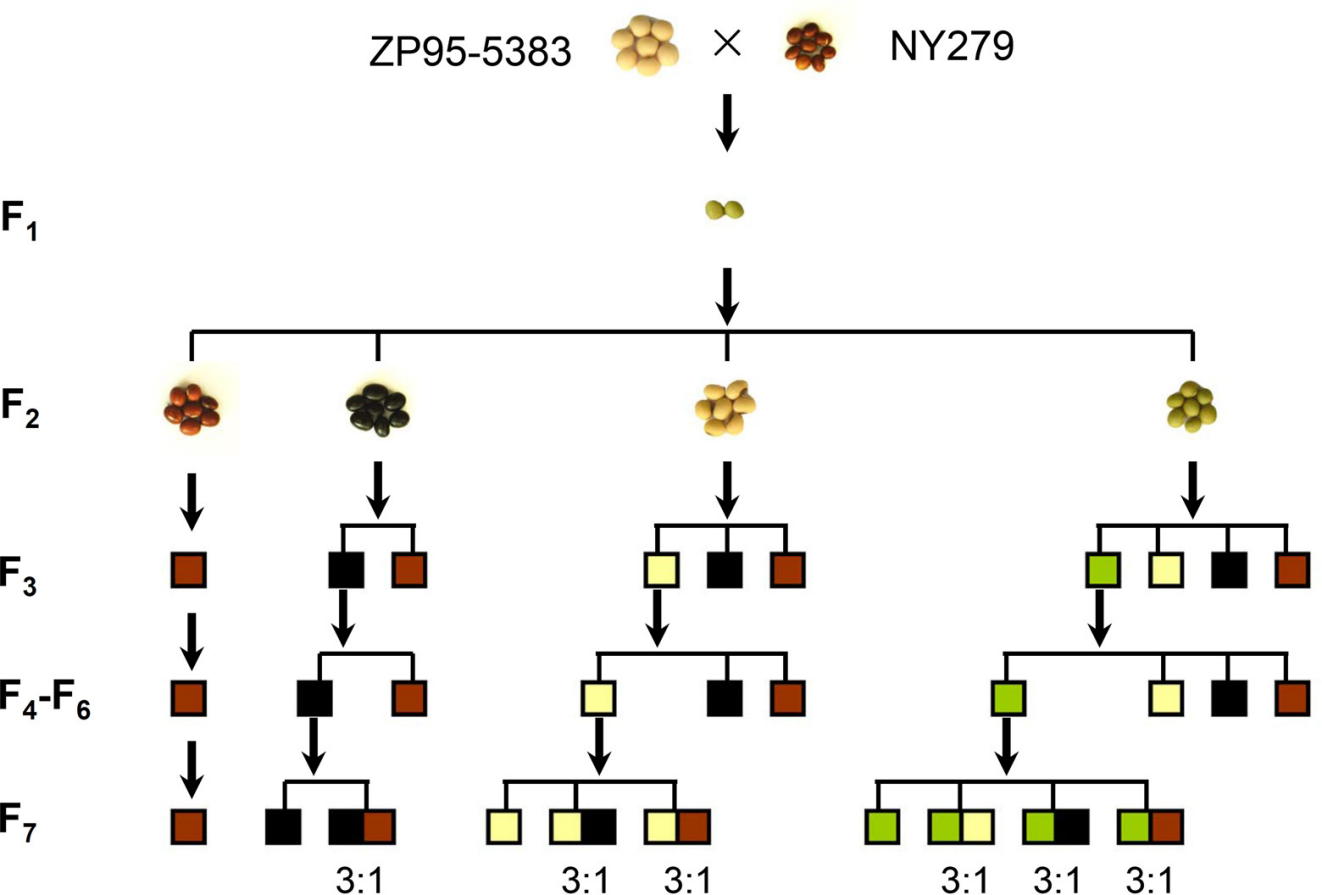
The inheritance of seed coat color in a segregating population derived from the cross between ZP95-5383 and NY279. Squares with different colors (green, yellow, black, and brown) represented soybeans with corresponding seed colors. Similar pattern of inheritance from soybeans with black, yellow, green seed in F_3_-F_7_ generation were not shown completely in this diagram.

To confirm the candidate loci identified in association mapping, a biparental population derived from the cross between ZP95-5383 (yellow seed coat) and NY279 (brown seed coat) was used. Seed coat color of F_1_ plant was green and four different colors were observed in F_2_ generation (30, 109, 17, and 15 individuals showed yellow, green, brown and black seed coat separately). Genetic analysis of different generations from F_2_ to F_7_ revealed that brown seed coat did not segregate at all while individuals with black seed only generated progeny with black or brown seed. Some individuals possessing yellow seed coat could generate soybeans with yellow, black, and brown colors and the segregation of green seed coat was just like that in F_1_ generation (Fig 4).

Bulk segregation analysis was carried out using 33 polymorphic SSR markers (S3 Table) near fourteen associated loci. DNA bulks from F_2_ individuals with green, yellow, black, and brown seed were first screened with polymorphic markers. The results suggested that only two loci cosegregated with specific colors of bulks. Four markers located in *qSC1* region co-segregated with yellow seed coat and three markers in *qSC5* region co-segregated with black and brown.

To further dissect other loci controlling seed coat color in this segregating population, several RHLs were developed for different color pairs including green/yellow, green/black, green/brown, yellow/black, yellow/brown, black/brown after phenotypic selection and self-fertility for several generations (Fig 4). DNA bulks of different color pairs from these RHL populations were also identified with polymorphic markers. Similar to the results from F_2_ individuals, markers in *qSC1* region co-segregated with color pair of green/yellow and markers in *qSC5* co-segregated with green/black, green/brown, yellow/black, yellow/brown. However, three markers located in *qSC2* region and three markers in *qSC7* were all identified to co-segregate with color pair of black/brown in two different RHL populations, which was not detected from bulks of F_2_ individuals.

### Fine mapping of loci identified by combining association mapping and bulk segregation analysis

To further map these four loci of *qSC1;2;5;7*, individuals consisting of DNA pools were all genotyped with the polymorphic markers for each locus. The results revealed that all markers at every locus clearly co-segregated with the phenotype of different seed coat colors. Among them, *qSC1* was a novel one controlling green/yellow while the other three loci located at similar regions of *T*, *I*, and *R* loci. Using different RHL populations, *qSC1*, *qSC2*, *qSC5*, and *qSC7* were successfully mapped between markers BARCSOYSSR_1_1503 and 1_1546, 6_942 and 6_998, 8_459 and 8_480, and Sat_352 and Satt196, respectively (Table 3).

**Table 3:** Details of loci governing seed coat color identified via BSA in soybean.

Locus	Chr.	Associated phenotype	Genetic region	Physical position (assembly v1.1)	Physical position (assembly v2.0)	Physical distance (kb)
*qSC1*	1	Green/Yellow	1_1503~1_1546	51,910,240~52,633,016	52,797,517~53,517,890	723
*qSC2/T*	6	Black/Brown	6_942~6_998	17,443,860~18,713,575	17,498,347~18,918,726	1,270
*qSC5/I*	8	Green/Black, Green/Brown, Yellow/Black, Yellow/Brown	8_459~8_480	8,321,840~8,745,942	8,326,164 ~8,775,965	424
*qSC7/R*	9	Black/Brown	Sat_352~Satt196	41,890,948~43,310,941	45,087,036~46,515,708	1,420

**Fig 5:**
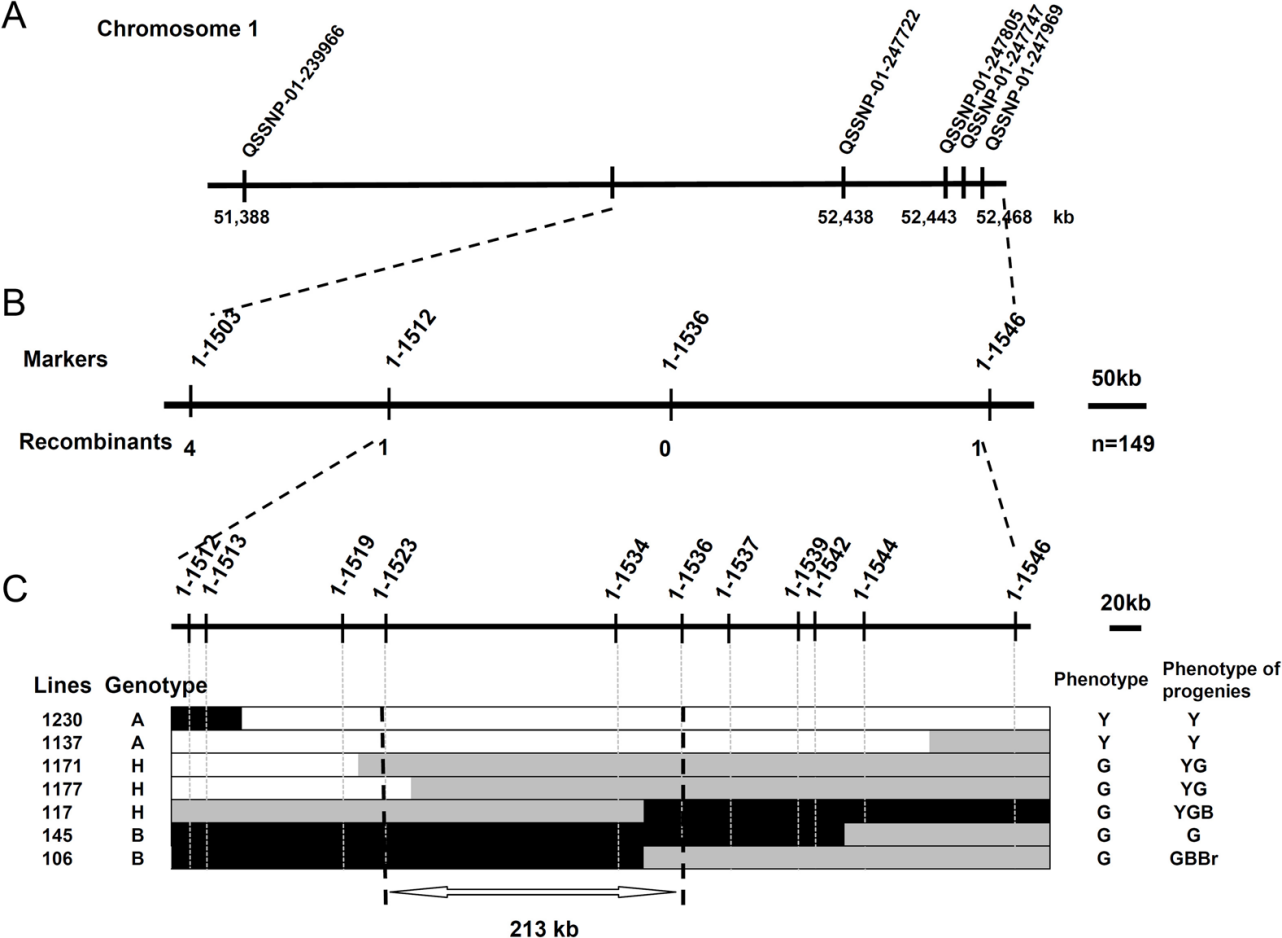
Fine mapping of *qSC1* locus. (A) Chromosomal location of *qSC1* identified by association mapping on chromosome 1. The significant associated SNPs were indicated above the line. (B) Roughly mapping of *qSC1* by using RHL populations. Vertical lines represented polymorphism markers. The names of markers and the number of recombinants between *qSC1* and each marker were shown above and below the line separately. (C) Fine mapping of *qSC1* locus by detailed marker-phenotype analysis of recombinants. The genotype of each recombinant was confirmed based on the phenotypes of its progeny. The black/gray/white colors indicated homozygousity/heterozygousity/homozygousity of markers based on genotypes of parental lines and the delimited region for the *qSC1* locus is indicated by bold arrow. Y (Yellow), G (Green), B (Black), and Br (Brown) represented different seed colors of recombinants and their progeny. All the physical positions of markers were according to assembly v1.1 of soybean genome.

Since genes corresponding to *I*, *T* and *R* loci have been identified in the previously reports [23–26, 30], selected RHL populations were used for fine mapping of *qSC1* locus. Eleven polymorphic markers between BARCSOYSSR_1_1503 and 1_1546 were developed and subsequent marker-phenotype analysis enabled us to refine *qSC1* region into a 213-kb interval (23 candidate genes) between markers BARCSOYSSR_1_1523 and 1_1536 (Fig 5).

### Molecular marker development and the interaction of different loci

Combinations of different loci can be used to infer genetic effect of each locus for specific trait. Three SSR markers closely linked to *qSC1*, *qSC5/I*, *qSC7/R* loci (BARCSOYSSR_1_1528, 8_466, and 9_1491) and a dCAPS marker of *GmF3’H* gene for *qSC2/T* locus were used for genotyping entire F_2_ population. The results revealed that all individuals possessing dominant *qSC5/I* allele showed green or yellow seed coats while soybeans with recessive *qsc5/i* allele showed black or brown coats. In the *qSC5/I* background, seed colors of all individuals possessing dominant *qSC1* allele were green while soybeans with recessive *qsc1* allele showed yellow seed coats. Furthermore, seed colors of individuals with recessive *qsc2/t* locus in the *qsc5/i* background were brown. However, when individuals possessed dominant allele of *qSC2/T* and recessive allele of *qsc5/i*, *qSC7/R* locus could be used for distinguishing black and brown seed coat (Table 4). From these results the interaction of different loci can be concluded, in which *qSC5/I* locus controlled pigmentation of seed coat to dark colors and *qSC1* governed further pigmentation of relative light color on the basis of *qSC5/I* locus. In addition, *qSC2/T* and *qSC7/R* loci were responsible for pigmentation of different degrees of dark colors and *qSC2/T* locus might function upstream of *qSC7/R* in this network.

**Table 4:** The relationship of genotypes and seed coat colors in F_2_ segregating population.

Genotype				No. of individuals			
				Green	Yellow	Black	Brown
*qSC5/I*^a^	*qSC1*	*qSC2/T*	*qSC7/R*	57	0	0	0
*qSC5/I*	*qSC1*	*qSC2/T*	*qsc7/r*	16	0	0	0
*qSC5/I*	*qSC1*	*qsc2/t*	*qSC7/R*	26	0	0	0
*qSC5/I*	*qSC1*	*qsc2/t*	*qsc7/r*	10	0	0	0
*qSC5/I*	*qsc1*	*qSC2/T*	*qSC7/R*	0	15	0	0
*qSC5/I*	*qsc1*	*qSC2/T*	*qsc7/r*	0	4	0	0
*qSC5/I*	*qsc1*	*qsc2/t*	*qSC7/R*	0	8	0	0
*qSC5/I*	*qsc1*	*qsc2/t*	*qsc7/r*	0	3	0	0
*qsc5/i*^b^	*qSC1*	*qSC2/T*	*qSC7/R*	0	0	12	0
*qsc5/i*	*qsc1*	*qSC2/T*	*qSC7/R*	0	0	3	0
*qsc5/i*	*qSC1*	*qSC2/T*	*qsc7/r*	0	0	0	2
*qsc5/i*	*qsc1*	*qSC2/T*	*qsc7/r*	0	0	0	0
*qsc5/i*	*qSC1*	*qsc2/t*	*qSC7/R*	0	0	0	8
*qsc5/i*	*qsc1*	*qsc2/t*	*qSC7/R*	0	0	0	1
*qsc5/i*	*qSC1*	*qsc2/t*	*qsc7/r*	0	0	0	3
*qsc5/i*	*qsc1*	*qsc2/t*	*qsc7/r*	0	0	0	3

^a^The uppercase letter of locus symbol indicated dominant or heterozygous alleles.

^b^The lowercase letter of locus symbol indicated recessive allele.

## Discussion

### Combination of association mapping and biparental mapping enhance the mapping resolution

Association mapping has been proven to be a powerful tool to identify loci associated with important traits even at single gene resolution in *Arabidopsis*, rice and maize [45–47]. In soybean, only hundreds of SSR markers or few thousands of SNPs have been used in association analysis at the early stage [65–69]. However, the marker density was too low to detect QTLs powerfully, resulting in difficult isolation of genes. A couple of recent reports have increased markers to several thousands or tens of thousands with GBS (genotyping by sequencing) or SNP chips, but the resolution is still not very high because of the long-range LD (linkage disequilibrium) [51, 70–72]. In addition, it is likely that contributions of coding SNPs to phenotypic variations would be higher than SNPs in non-coding regions [73]. Therefore, association analysis with SNPs in coding regions may get more specific results compared to SNPs in non-coding regions. Moreover, our results also indicated that non-synonymous and synonymous coding SNPs have similar effects on association mapping.

Association and biparental mapping have complementary advantages and disadvantages and their limitations could be mitigated by using both analysis [34]. The combination of these two approaches has been employed in model plants and successful isolation of gene for QTL has proven the usefulness of this strategy [74, 75]. A locus having an effect in multiple accessions could be detected in association mapping while only loci harboring major effects can be mapped in a biparental population [76]. Therefore, once a trait is correlated with the structure of a natural population, the power of association analysis is reduced, whereas biparental mapping can be used to detect QTLs in a population derived from accessions belonging to different subgroups [50, 77]. Therefore, identification of QTLs using both biparental and association mapping in the same study will provide more robust understanding of genetic architecture than any single method. In this study, although a total of 14 loci were identified in association mapping, only four of them were confirmed by BSA in the biparental population used. The other loci may be validated using other segregating populations.

### Comparison of identified loci with previously reported QTLs

Seed coat color is not only related to biochemical functions of secondary metabolism, antioxidant activity, and disease resistance but also a morphological trait for classification of germplasm and evolutionary analysis [3–5]. Apart from five genetic loci controlling flavonoid-based pigmentation [13, 14], eight QTLs on five chromosomes have also been identified through QTL mapping but mapping regions were always too large due to limited number of markers used [78–80]. Among them, two QTLs (seed coat color2-1 and 3–1) were all close to *I* locus, but it was difficult to confirm whether they were the same QTL due to the large genomic regions in their studies. Moreover, some reports also revealed that combination of gene-based markers of *T* and *W1* loci or two SNP outliers could partially increase selection efficiency for seed colors [64, 81]. Therefore, soybean seed color has a relative complex genetic basis and accessions with same color possibly having different genotypes at these loci.

All five loci identified previously were detected in our results of association mapping, suggesting the representative of soybean accessions used. Meanwhile, all associated SNPs at 14 loci could separate soybeans with different colors more properly than only using five previous reported loci (Fig 3), further indicating the accuracy of our association analysis. Moreover, four loci including a novel one were confirmed by biparental mapping, indicating that we identified common or major loci in both natural and biparental populations. Eight of the rest ten loci could be further confirmed using segregating populations developed from other accessions because our biparental population even did not detect *W1* and *O* loci. In addition, previous studies also revealed that *G* locus linked with *D1* was mapped to LG D1a (chromosome 1) of soybean but no detailed information of physical position [82, 83]. Cloning and characterization of *D1* supported that *Glyma01g42390* is *D1* controlling stay-green in soybean [32, 33]. Therefore, *qSC1* on chromosome 1 may be considered as *G* locus and the fine mapping region of 213kb will be useful for map-based cloning of *G* gene.

### Systematic dissection of complex trait as a powerful tool for discovering genes

Even though a high quality and well annotated genome sequence has been available [63, 84], isolation of genes for QTLs is still somewhat difficult in soybean. Majority of QTL mapping studies in soybean using hundreds of molecular markers with population size of a few hundred always identified dozens or even hundreds of QTLs (http://www.soybase.org). However, few of these QTLs are common to all mapping efforts. Moreover, the difficult of developing NILs in soybean further restrict the usage of this approach for fine mapping of QTLs. Development of RHLs is another choice for evaluating QTLs in soybean since some relative complex traits could be divided into several simple trait pairs in RHL populations [42, 43, 85]. In this study, four kinds of seed colors in segregating population were dissected into six simple color pairs by continuous self-fertility and selection of progeny. Finally all four QTLs were identified and validated by using these RHL populations and markers located in associated regions.

When BSA method was used to confirm loci identified in association analyses, only two major loci (*qSC1* and *qSC5/I*) were confirmed from bulks of F_2_ individuals. After continuously developing RHLs, another two loci (*qSC2/T* and *qSC7/R*) were further identified. These four loci explained all genetic variations of seed coat colors in this segregating population, indicating that the strategy of systematically dissecting relative complex trait to simple trait pairs could serve as a powerful approach for discovering multiple genes which may have little effect.

### The interaction of different loci controlling seed coat color

Previous reports indicated that *I* locus had major effect on controlling pigmentation of seed coat [14, 21, 22]. Our results from association mapping, BSA of F_2_ individuals and RHL populations all supported this conclusion as *qSC5/I* locus could be used for distinguishing dark and light colors of seed coat. Since the seed colors of wild soybeans and modern cultivars are mainly black and yellow respectively, *qSC5/I* locus may undergo selection during soybean domestication. Previous report on resequencing of wild and cultivated soybeans also indentified three genes in *qSC5/I* region with strong selection signals [7]. *qSC1* locus was proven to co-segregate with green color and dominant *qSC1* allele could pigment light green color in *qSC5/I* allele background. Up to now, few reports illustrated the genetic basis of green seed color in soybean, partially because the segregation of this kind of individuals is more complex than others. There is also a possibility that the green color is fading out at maturity of soybean seed and becoming yellow under the control of *v1* or *g1* locus [17, 86]. Since *qSC5/I* was proven to regulate the expression of *CHS* genes which had function in early step in flavonoids and anthocyanins biosynthesis [23, 24, 87, 88], we postulated that *qSC1* might affect the coloration of seed coat in an independent pathway.

Furthermore, previous studies also revealed that *T* and *R* loci were associated with black and brown seed coats [14–16, 31]. Characterization of *R* locus suggested that functional *R* gene acted to promote transcription of structural genes encoding U3FGT and ANS which were located in downstream of flavonoid 3’-hydroxylase (encoded by *GmF3’H* gene) in anthocyanin pathway [30]. In this study, *qSC7/R* locus could be used for distinguish black and brown seed coats only under the background of dominant *qSC2/T* locus, also indicating that *qSC7/R* locus was involved in the downstream of *qSC2/T*. Therefore, further fine mapping and cloning of *qSC1* will contribute to construct regulatory network of seed coat pigmentation in soybean.

## Conclusions

**Fig 6:**
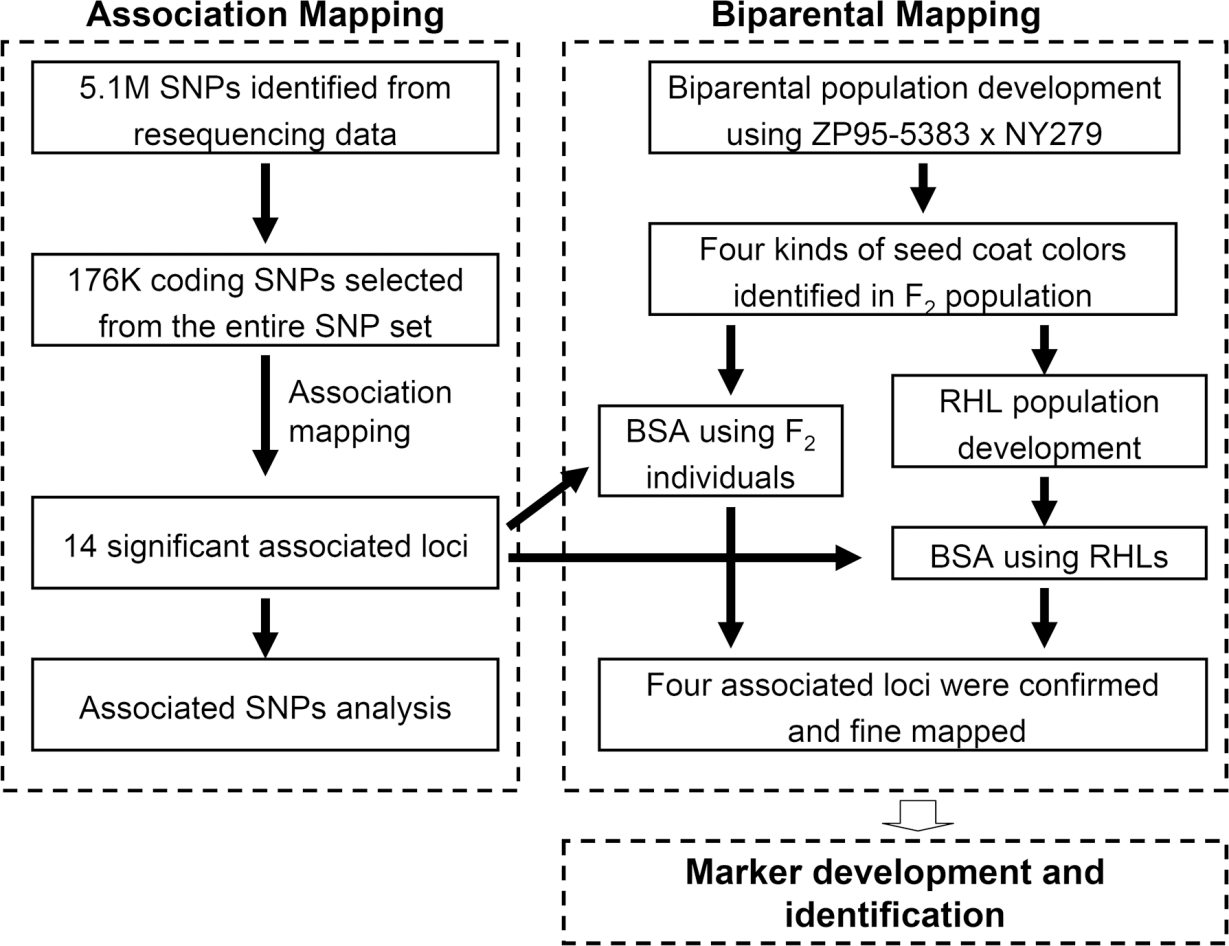
Flowchart of the approach to combine association and biparental mapping. Results of association mapping and bulk segregation analysis were summarized side by side to clearly describe the entire study.

A total of 14 loci distributed across ten chromosomes were identified to be associated with soybean seed coat colors using coding SNPs among a natural population. These loci could distinguish all tested soybean accessions with different colors more properly than five previous reported loci. Four of them including one novel locus were confirmed using several RHLs derived from a biparental population. The moderately complex trait of seed coat color was divided into simple color pairs and all four QTLs controlled this trait were systematically dissected by bulk segregation analysis and fine mapping (Fig 6). Even more, the regulation mechanism of these four loci was illustrated by genotyping entire F_2_ population using flanking markers of them. The results exhibiting in the manuscript could provide in-depth understanding of the inheritance of seed coat color and domestication analysis of different loci in soybean. The genetic information of these loci was useful for map-based cloning as well as marker-assisted selection in breeding program. Moreover, this work also provide an alternative strategy for systematically discovering genes by association analysis with high-throughput sequence data in natural population following bulk segregation analysis among dissected segregating populations.

## Supporting Information

S1 Fig Population structure of 56 soybean accessions. (A) Estimated ln (probability of the data) calculated for K ranging from 2 to 9. (B) Population structure of soybean accessions, each accession was represented by a single vertical line and every color represented one cluster. The red color indicated Subgroup I and the green color indicated subgroup II.
(PDF)

S2 Fig **Association mapping of seed coat color in soybean with SNPs in Sets B and C.** (A) Expect -log (*P*) matched observed -log (*P*) best from the QQ-Plot using SNPs from Set B. (B) Manhattan plots showed -log(*P*) from a genome-wide scan were plotted against positions of SNPs on 20 chromosomes using SNPs from Set B. (C) Expect -log (*P*) matched observed -log (*P*) best from the QQ-Plot using SNPs from Set C. (D) Manhattan plots showed -log(*P*) from a genome-wide scan were plotted against positions of SNPs on 20 chromosomes using SNPs from Set C.
(PDF)

S3 Fig Graphical representation of most significant associated SNPs in all 14 loci for 56 soybean accessions. Red represented allele of each locus present in the reference genome (Williams 82) and blue represented the alternate allele. In addition, green represented the heterozygous alleles and grey represented missing data.
(PDF)

S1 Table The general information of accessions used in this study. (PDF)

S2 Table Comparative analysis of the association mapping results using SNPs from Sets A, B, and C.
(PDF)

S3 Table Information of SSR markers near fourteen loci identified by association mapping. (PDF)
