# Supplementary material for: Identification and Validation of Loci Governing Seed Coat Color by Combining Association Mapping and Bulk Segregation Analysis in Soybean
Source: PLoS One. 2016 Jul 12;11(7):e0159064. doi: 10.1371/journal.pone.0159064 (PMC4942065; doi:10.1371/journal.pone.0159064)
Supplement: S1 Fig — (A) Estimated ln (probability of the data) calculated for K ranging from 2 to 9. (B) Population structure of soybean accessions, each accession was represented by a single vertical line and every color represented one cluster. The red color indicated Subgroup I and the green color indicated subgroup II. (PDF) [file pone.0159064.s001.pdf]

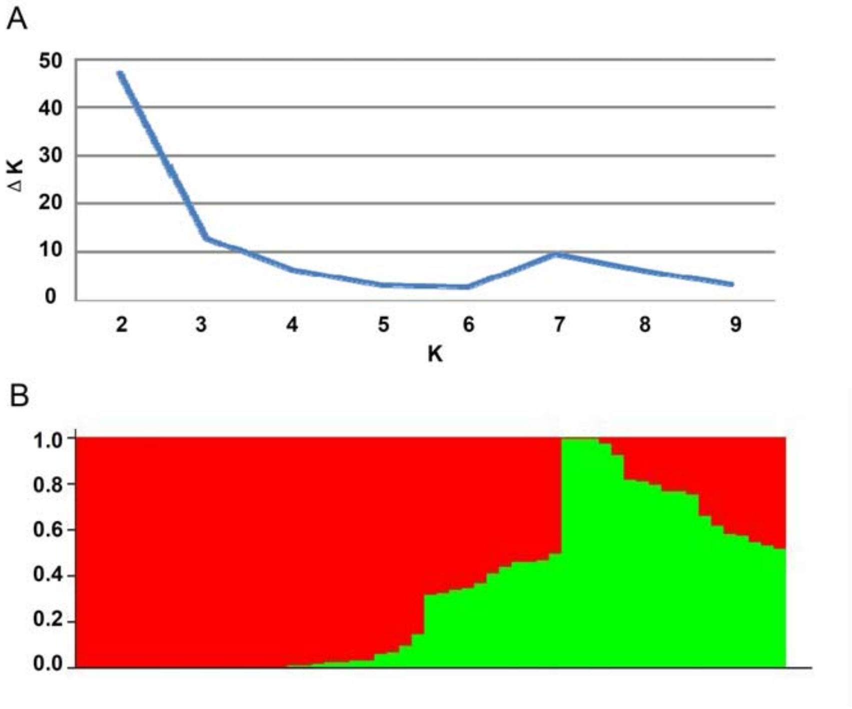

Figure S1 Population structure of 56 soybean accessions

(A) Estimated ln (probability of the data) calculated for K ranging from 2 to 9. (B) Population structure of soybean accessions, each accession was represented by a single vertical line and every color represented one cluster. The red color indicated Subgroup I and the green color indicated subgroup II.
