# Supplementary material for: Identification and Validation of Loci Governing Seed Coat Color by Combining Association Mapping and Bulk Segregation Analysis in Soybean
Source: PLoS One. 2016 Jul 12;11(7):e0159064. doi: 10.1371/journal.pone.0159064 (PMC4942065; doi:10.1371/journal.pone.0159064)
Supplement: S2 Fig — Association mapping of seed coat color in soybean with SNPs in Sets B and C. (A) Expect -log (P) matched observed -log (P) best from the QQ-Plot using SNPs from Set B. (B) Manhattan plots showed -log(P) from a genome-wide scan were plotted against positions of SNPs on 20 chromosomes using SNPs from Set B. (C) Expect -log (P) matched observed -log (P) best from the QQ-Plot using SNPs from Set C. (D) Manhattan plots showed -log(P) from a genome-wide scan were plotted against positions of SNPs on 20 chromosomes using SNPs from Set C. (PDF) [file pone.0159064.s002.pdf]

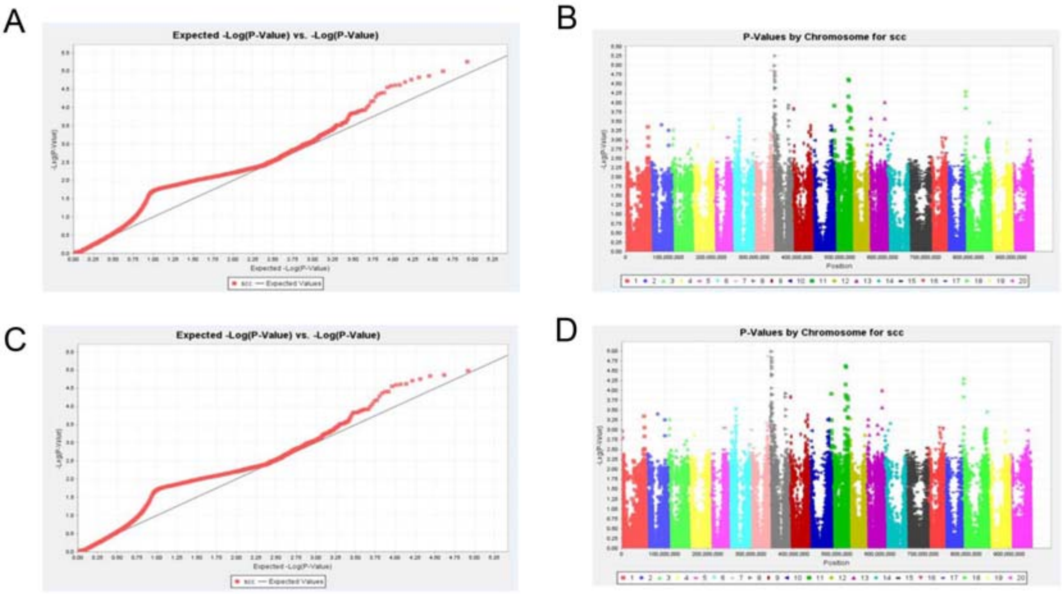

S2 Fig. Association mapping of seed coat color in soybean with SNPs in Sets B and C.

(A) Expect -log (P) matched observed -log (P) best from the QQ-Plot using SNPs from Set B. (B) Manhattan plots showed -log(P) from a genome-wide scan were plotted against positions of SNPs on 20 chromosomes using SNPs from Set B. (C) Expect -log (P) matched observed -log (P) best from the QQ-Plot using SNPs from Set C. (D) Manhattan plots showed -log(P) from a genome-wide scan were plotted against positions of SNPs on 20 chromosomes using SNPs from Set C.
