# Supplementary material for: Identification and Validation of Loci Governing Seed Coat Color by Combining Association Mapping and Bulk Segregation Analysis in Soybean
Source: PLoS One. 2016 Jul 12;11(7):e0159064. doi: 10.1371/journal.pone.0159064 (PMC4942065; doi:10.1371/journal.pone.0159064)
Supplement: S3 Fig — Red represented allele of each locus present in the reference genome (Williams 82) and blue represented the alternate allele. In addition, green represented the heterozygous alleles and grey represented missing data. (PDF) [file pone.0159064.s003.pdf]

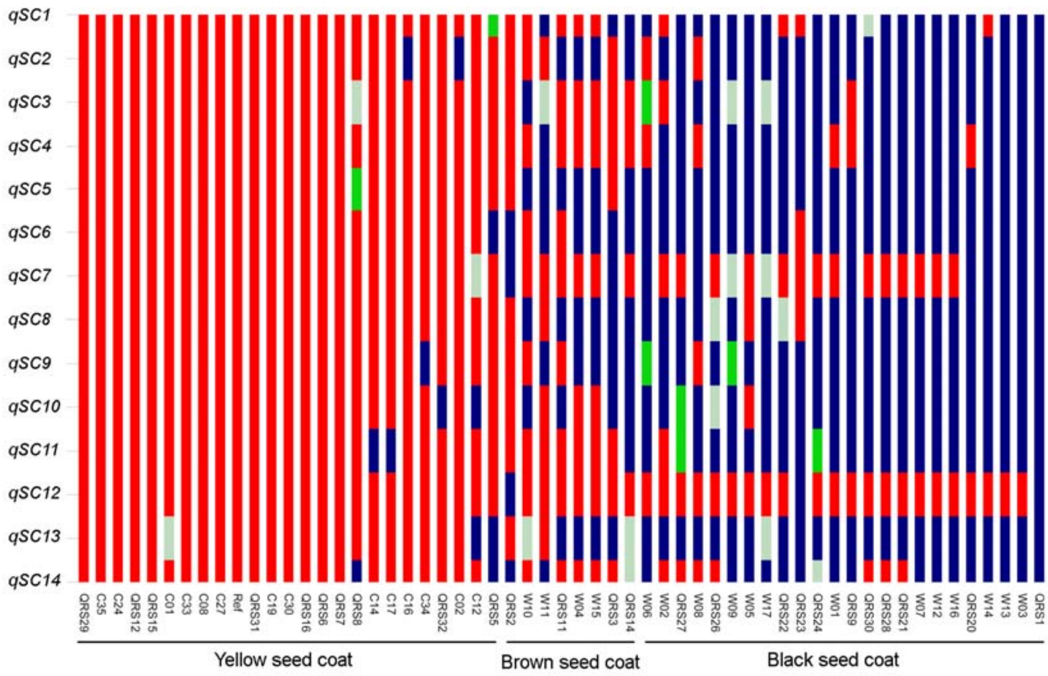

S3 Fig. Graphical representation of most significant associated SNPs in all 14 loci for 56 soybean accessions.

Red represented allele of each locus present in the reference genome (Williams 82) and blue represented the alternate allele. In addition, green represented the heterozygous alleles and grey represented missing data.
