# Supplementary material for: Identification and Validation of Loci Governing Seed Coat Color by Combining Association Mapping and Bulk Segregation Analysis in Soybean
Source: PLoS One. 2016 Jul 12;11(7):e0159064. doi: 10.1371/journal.pone.0159064 (PMC4942065; doi:10.1371/journal.pone.0159064)
Supplement: S1 Table — (PDF) [file pone.0159064.s004.pdf]

S1 Table. The general information for accessions used in this study

Code Name	Name of Accessions	Type of Accessions	Seed Coat Color
Ref	Williams82	Reference	Yellow
QRS1	Hanyuanbalixiaoheidou	Landrace	Black
QRS2	Yingdehedou	Landrace	Brown
QRS3	Changshanidou	Landrace	Brown
QRS5	Baiqiu1	Breeding line	Yellow
QRS6	Suinong14	Breeding line	Yellow
QRS7	Suinong20	Breeding line	Yellow
QRS8	Zhongpin03-5373	Breeding line	Yellow
QRS9	Huipizhiheidou	Landrace	Black
QRS11	Zhechengxiaohongdou	Landrace	Brown
QRS12	Pixiansilizao	Landrace	Yellow
QRS14	Xiataizimoshidou	Landrace	Brown
QRS15	Zheng92116	Breeding line	Yellow
QRS16	Zhonghuang13	Breeding line	Yellow
QRS20	Heidou	Landrace	Black
QRS21	ZYD03687	Wild soybean	Black
QRS22	ZYD00401	Wild soybean	Black
QRS23	ZYD04186	Wild soybean	Black
QRS24	ZYD02878	Wild soybean	Black
QRS26	ZYD04734	Wild soybean	Black
QRS27	ZYD04569	Wild soybean	Black
QRS28	ZYD04638	Wild soybean	Black
QRS29	Shang951099	Breeding line	Yellow
QRS30	ZYD02738	Wild soybean	Black
QRS31	Ji-NF58	Breeding line	Yellow
QRS32	Jidou12	Breeding line	Yellow
C01	Wenfeng7	Breeding line	Yellow
C02	Tiefeng8	Breeding line	Yellow
C08	Union	Breeding line	Yellow
C12	Jindou21	Breeding line	Yellow
C14	Brazil10	Breeding line	Yellow
C16	Tainong1	Breeding line	Yellow
C17	Zigongdongdou	Landrace	Yellow
C19	Jilinxiaoli	Landrace	Yellow
C24	Gandou4	Breeding line	Yellow
C27	Cangdou5	Breeding line	Yellow
C30	Yudou12	Breeding line	Yellow
C33	Hefeng25	Breeding line	Yellow
C34	Gui199	Landrace	Yellow
C35	Guangzhoudali	Landrace	Yellow
W01	Beijing4	Wild soybean	Black

W02	Donggou16	Wild soybean	Black
W03	Wuhai4	Wild soybean	Black
W04	Yanjin3	Wild soybean	Brown
W05	Mengjin1	Wild soybean	Black
W06	Jidong5	Wild soybean	Black
W07	Fengcheng20	Wild soybean	Black
W08	Jixian11	Wild soybean	Black
W09	Kaiyuan21	Wild soybean	Black
W10	Shuangcheng4	Wild soybean	Brown
W11	Taiyuan67	Wild soybean	Brown
W12	Anqing18	Wild soybean	Black
W13	Yimeng15	Wild soybean	Black
W14	Yimeng19	Wild soybean	Black
W15	Yanjin14	Wild soybean	Brown
W16	Hailun19	Wild soybean	Black
W17	Zhangwu20	Wild soybean	Black
