# Supplementary material for: Identification and Validation of Loci Governing Seed Coat Color by Combining Association Mapping and Bulk Segregation Analysis in Soybean
Source: PLoS One. 2016 Jul 12;11(7):e0159064. doi: 10.1371/journal.pone.0159064 (PMC4942065; doi:10.1371/journal.pone.0159064)
Supplement: S2 Table — Comparative analysis of the association mapping results using SNPs from Sets A, B, and C. (PDF) [file pone.0159064.s005.pdf]

S2 Table. Comparative analysis of the association mapping results using SNPs from Sets A, B, and C

Locus	Chr.	Physical position (assembly v1.1)	Physical position (assembly v2.0)	Range(kb)	Number of associated SNPs in Set A	Number of associated SNPs in Set B	Number of associated SNPs in Set C
qSC1	1	51,388,129-52,467,583	52,275,421-53,353,044	1,079	5	2	2
qSC2	6	18,878,314-19,047,336	19,082,159-19,251,651	169	7	5	4
qSC3	7	41,940,392-43,082,019	41,886,767-43,027,411	1,142	6	4	3
qSC4	8	3,417,779-5,512,389	3,420,044-5,519,599	2,095	6	4	4
qSC5	8	6,783,439-8,648,879	6,789,359-8,678,906	1,865	51	29	26
qSC6	8	39,473,028-40,585,746	40,107,160-41,211,537	1,113	8	5	5
qSC7	9	38,277,760-43,419,283	40,878,957-46,624,175	5,142	5	5	5
qSC8	10	42,771,760-43,820,987	43,338,444-44,401,056	1,049	15	3	2
qSC9	11	681,423-1,055,084	690,398-1,064,066	374	9	5	2
qSC10	11	38,370,581-38,636,896	33,897,357-34,160486	266	12	7	6
qSC11	12	5,404,396-5,649,464	5,412,697-5,660,796	245	3	1	1
qSC12	13	6,661,165-7,743,106	14,111,454-15,225,051	1,082	9	4	3
qSC13	13	39,045,030-39,157,835	40,223,970-40,336,776	113	5	4	3
qSC14	18	57,910,138-57,962,686	53,640,208-53,693,058	53	5	2	2
