# Supplementary material for: Identification and Validation of Loci Governing Seed Coat Color by Combining Association Mapping and Bulk Segregation Analysis in Soybean
Source: PLoS One. 2016 Jul 12;11(7):e0159064. doi: 10.1371/journal.pone.0159064 (PMC4942065; doi:10.1371/journal.pone.0159064)
Supplement: S3 Table — (PDF) [file pone.0159064.s006.pdf]

S3 Table. Information of SSR markers near fourteen loci identified by association mapping

Maker Name	BARCSOYSSR Sequence ID	Primer	Sequences	Product size(bp)	Physical Position (assembly v1.1)	Physical Position (assembly v2.0)	Locus
1-1536	Barcsoyssr_1_1536	Forward	AATCCAAAATCCTGTCGTGC	217	52,438,759	53,323,483	*qSC1*
		Reverse	CATTCACACAAGCTGCAACA		52,438,784	53,323,508	
1-1528	Barcsoyssr_1_1528	Forward	TCTTAAGATTTGCCCCGTTG	270	52,316,259	53,203,363	*qSC1*
		Reverse	GGCTTTTACAAAATATTGAAGTTTGA		52,316,312	53,203,416	
1-1544	Barcsoyssr_1_1544	Forward	TTGGATCATGTCAATTTCTTTTT	239	52,530,918	53,415,809	*qSC1*
		Reverse	AAAAGTTGGACCAAACGGGT		52,530,949	53,415,840	
1-1546	Barcsoyssr_1_1546	Forward	ATGCCGCTTCTCATTCAGAT	210	52,632,975	53,517,849	*qSC1*
		Reverse	TTGATGATTTAGGTTACGGTGC		52,633,016	53,517,890	
6-998	Barcsoyssr_6_998	Forward	TCAAAACAATAATTTTCGAGGAA	288	18,713,522	-	*qSC2*
		Reverse	TGTAATTATTTTGAGTCGAAGGAAG		18,713,575	-	
6_1032	Barcsoyssr_6_1032	Forward	GGATGAGTTTGATAAACATGAATGA	273	19,653,852	19,858,118	*qSC2*
		Reverse	TTATTTGGAGCCCTCCCCT		19,653,932	19,858,198	
Satt557	Barcsoyssr_6_1041	Forward	GCGGGATCCACCATGTAATATGTG	207	20,018,845	20,218,992	*qSC2*
		Reverse	GCGCACTAACCCTTTATTGAA		20,018,907	20,219,054	
Satt308	Barcsoyssr_7_1556	Forward	GCGTTAAGGTTGGCAGGGTGGAAGTG	170	42,876,591	42,822,341	*qSC3*
		Reverse	GCGCAGCTTTATACAAAAATCAACAA		42,876,656	42,822,406	
7-1552	Barcsoyssr_7_1552	Forward	TGTCCATCAGGCGTCAGTAA	231	42,805,187	42,750,937	*qSC3*
		Reverse	CGTGGAGATCAAAGTTGGGT		42,805,237	42,750,987	
Sat_276	Barcsoyssr_7_1561	Forward	GCGGAAACCCATCTAGAATATGAAAAACA	272	42,916,026	42,861,774	*qSC3*
		Reverse	GCGTTCTTCTCGAGGTGAGATACAATC		42,916,087	42,861,835	
8-248	Barcsoyssr_8_248	Forward	TGTCACCATTTCAATCCTAATCA	289	4,726,529	4,732,715	*qSC4*
		Reverse	CTCCCATTCAACATCAGCAG		4,726,596	4,732,782	

8-250	Barcsoyssr_8_250	Forward	TGCTGCATTTGGCATTACAT	261	4,760,029	4,766,186	*qSC4*
		Reverse	TCATTTTTCTCACTTTGGTCCC		4,760,064	4,766,221	
Satt493	Barcsoyssr_8_262	Forward	GTTAACGGGAAAAAATTAAACCTA	251	5,027,329	5,033,482	*qSC4*
		Reverse	GTGGCTGCCTAGTGCTGTTTTATA		5,027,364	5,033,517	
8-459	Barcsoyssr_8_459	Forward	CAACTTTCGCATCGGTTACA	232	8,321,840	8,326,164	*qSC5*
		Reverse	CGGAAACTGCTTATGGTTGC		8,321,883	8,326,207	
8-466	Barcsoyssr_8_466	Forward	TGACATGGCGTGATTGTTCT	224	8,423,496	8,438,244	*qSC5*
		Reverse	ACACATCCAGTCCCAGGAAA		8,423,563	8,438,311	
8-475	Barcsoyssr_8_475	Forward	TTAAAGAGCCTTACATGACATCTG	286	8,613,309	8,643,336	*qSC5*
		Reverse	CCGTATTCAAAATTGACTAGCAT		8,613,368	8,643,395	
Sat-352	Barcsoyssr_9_1453	Forward	GCGTATTAGACTAGTGCTATTCGCGAGTTA	224	41,890,948	45,087,036	*qSC7*
		Reverse	GCGTGTCATTTCATTAAAAACAAAAACATAA		41,891,009	45,087,097	
Sat-293	Barcsoyssr_9_1512	Forward	GCGTTAGGCAAATGAGATGTCAA	281	42,848,842	46,053,189	*qSC7*
		Reverse	GCGCAGGGCAGTCATCGGAGGTAT		42,848,903	46,053,250	
Satt196	Barcsoyssr_9_1541	Forward	TTGGGAAATAGTGATTGAGGTAAAA	189	43,310,876	46,515,643	*qSC7*
		Reverse	AAATCCCCATTGAATGAGAATAAG		43,310,941	46,515,708	
Satt592	Barcsoyssr_10_1339	Forward	GCGAAGATTGGTCTTTTATGTCAAATG	241	42,983,859	43,509,853	*qSC8*
		Reverse	GCGGAGGAATACAAGTCTCTATTCAA		42,983,897	43,509,891	
10-1362	Barcsoyssr_10_1362	Forward	CCCCCTAAATATAGATCGCCT	238	43,451,401	44,031,058	*qSC8*
		Reverse	GGAACCAACCACACTCTCCA		43,451,442	44,031,099	
11-38	Barcsoyssr_11_38	Forward	AAGTAACACCCTCATGTGCAA	185	788,385	797,360	*qSC9*
		Reverse	GTGGTCAAAGCAAGTCGTGA		788,458	797,435	
11-46	Barcsoyssr_11_46	Forward	CCATCCATTCTTGTTGTGGA	173	1,062,244	1,071,226	*qSC9*
		Reverse	GCAATAGCCGTAGGTTTTGG		1,062,275	1,071,257	
Satt453	Barcsoyssr_11_1468	Forward	GCGGAAAAAAAACAATAAACAACA	234	38,360,612	34,173,223	*qSC10*
		Reverse	TAGTGGGGAAGGGAAGTTACC		38,360,653	34,173,264	

12-270	Barcsoyssr_12_270	Forward	CTCGTGGACTGATAAAGGGC	264	5,531,936	5,542,880	*qSC11*
		Reverse	GCTAATTACCCTTTTGTTCAGGG		5,532,005	5,542,949	
12-286	Barcsoyssr_12_286	Forward	ACGCCACCATCTCTCAAAGT	248	5,706,951	5,718,418	*qSC11*
		Reverse	CCCTGCTAGAAACAAGAACCC		5,707,018	5,718,485	
Satt269	Barcsoyssr_13_340	Forward	GCGTGCCAGGTAGAAAAATATTAG	253	6,580,533	15,306,342	*qSC12*
		Reverse	GCGGTTTTTCACTTTTCAAAATTC		6,580,565	15,306,374	
13-368	Barcsoyssr_13_368	Forward	TGACCCCATGTGTTCAAATTA	222	7,178,656	14,672,696	*qSC12*
		Reverse	AATGTGGATACCAAACATGGA		7,178,739	14,672,779	
Satt569	Barcsoyssr_13_458	Forward	GCGCAAATTGCTTCACGCATCCAAAT	184	9,567,219	12,294,094	*qSC12*
		Reverse	GCGGCCTACTATAGTGAAGGGTATA		9,567,260	12,294,135	
Satt193	Barcsoyssr_13_477	Forward	GCGTTTCGATAAAAATGTTACACCTC	239	10,032,901	11,847,952	*qSC12*
		Reverse	TGTTCGCATTATTGATCAAAAAT		10,032,969	11,848,020	
Satt472	Barcsoyssr_18_1708	Forward	GCGAATACATAAAACTCAAATTCAAATCATA	279	58,136,231	53,866,606	*qSC14*
		Reverse	GCGTTCTATAAATTTCATTCATAGTTTCAAT		58,136,341	53,866,716	
